# Epidemiology and Clinical Profile of Aspergillus Hypersensitivity and Allergic Bronchopulmonary Aspergillosis in Children with Asthma: A Hospital-Based Study

**DOI:** 10.4314/ejhs.v36i1.3

**Published:** 2026-01

**Authors:** Achal S Goindi, Jagroop S Sangha, Prabhpreet K Sira

**Affiliations:** 1 Department of Radiodiagnosis, Punjab Institute of Medical Sciences (PIMS), Jalandhar, Punjab; 2 Department of Paediatrics - JBMM District Hospital, Amritsar, Punjab; 3 Department of Paediatrics, Punjab Institute of Medical Sciences (PIMS), Jalandhar, Punjab

**Keywords:** Asthma, Allergy, Airway inflammation, Aspergillosis

## Abstract

**Background:**

The aim of this study was to examine the prevalence of Aspergillus sensitivity (AS) and allergic bronchopulmonary aspergillosis (ABPA) in children with Bronchial Asthma.

**Methods:**

The study included 200 children with bronchial asthma. ABPA was diagnosed using Aspergillus skin testing, pulmonary function testing, absolute eosinophil count, total serum IgE, Aspergillus-specific IgE and IgG, chest radiography, and high-resolution computed tomography. Patients were diagnosed and classified according to the Rosenberg–Patterson criteria for ABPA.

**Results:**

Of the 200 children with bronchial asthma, 7 patients were ABPA positive. Among the ABPA-positive cases, 5 (71.4%) had uncontrolled asthma. The mean absolute eosinophil count (AEC) was significantly higher in ABPA-positive cases (418.71 ± 90.12 cells/cumm). The mean total serum IgE in positive cases was 1181.00 ± 403.42 IU/L. The mean Aspergillus-specific IgE level in positive cases was 4.74 ± 6.09 kUA/L. In ABPA-positive cases, the mean Aspergillus-specific IgG level was 26.06 ± 14.93 mgA/L.

**Conclusion:**

This study demonstrated an association between ABPA and bronchial asthma in children. ABPA is a common entity in bronchial asthma and should be considered as a cause of uncontrolled asthma when symptoms persist despite the use of multiple medications.

## Introduction

Asthma is the most common chronic respiratory disease worldwide, with an estimated prevalence of 320 million patients (1). According to the latest GINA guidelines 2024, bronchial asthma is a heterogeneous disease, usually characterized by chronic airway inflammation. It is defined by a history of respiratory symptoms such as wheeze, shortness of breath, chest tightness, and cough that vary over time and in intensity, together with variable expiratory airflow limitation (2).

The diagnosis of bronchial asthma can be made if two out of the following three criteria are fulfilled: 1) History of recurrent attacks of chest tightness, breathlessness, and cough that is worse at night or early morning 2) Physical examination showing expiratory wheeze (rhonchi). 3) Spirometry showing airflow limitation or reduced FEV1 or FEV1: FVC < 0.80, or a bronchodilator response defined as an improvement in FEV1 of ≥12%/≥200 ml, or exercise challenge showing a worsening in FEV1 ≥15%(3).

Exacerbations, or asthma attacks, are major contributors to the burden of asthma and, although they may occur in any patient, are more frequent and severe in those with severe disease (1). Asthma exacerbations are episodes of progressive worsening of shortness of breath, cough, wheezing, or chest tightness, or a combination of these symptoms, requiring systemic corticosteroids or emergency department visits and hospital admission (5).

Asthma exacerbations may be precipitated by a variety of factors, ranging from allergens and occupational exposures to environmental and physiological triggers. Common allergens include house dust mite, pollens, animal dander, etc (4). A growing body of evidence from human and animal studies has demonstrated a link between fungal exposure, particularly indoor fungal exposure, and asthma initiation, persistence, and exacerbation (5). Airborne fungi such as Alternaria, Aspergillus, Cladosporium, and Penicillium are associated with asthma and other respiratory disorders. Among these, Aspergillus fumigatus is the most significant and is capable of causing a spectrum of conditions ranging from allergic reactions to chronic airway inflammation and severe aspergillosis, depending on host genetics and immune response (6).

This study assessed the prevalence of Aspergillus Sensitivity (AS) and Allergic Bronchopulmonary Aspergillosis (ABPA) in children with Bronchial Asthma and categorization of ABPA into various subgroups among the enrolled patients.

## Material and Methods

This study was conducted on a total of 200 children aged 1–15 years who were diagnosed with bronchial asthma. The study population included 136 outpatients and 64 inpatients from the Department of Paediatrics, Government Medical College and Raj indra Hospital, Patiala.

**Inclusion criteria**: Children aged 1–15 years with a diagnosis of bronchial asthma. The diagnosis of bronchial asthma was made if two out of the following three criteria were fulfilled: 1. History of recurrent attacks of chest tightness, breathlessness, and cough that is worse at night or early morning 2. Physical examination showing expiratory wheeze (rhonchi) 3. Spirometry showing airflow limitation or reduced FEV1 or FEVLFVC < 0.80, or bronchodilator response defined as an improvement in FEV1 of ≥12%/≥200 ml, or exercise challenge showing a worsening in FEV1 ≥15% (3).

**Exclusion criteria**: The following groups of children were excluded from the study-Children with congenital lung malformations, children whose families did not provide consent, immunocompromised children (primary or secondary), children with protein–energy malnutrition grade 3 or 4, asthmatic children receiving oral steroids, and children on medications other than anti-asthmatic drugs, such as those used for cardiac disease or tuberculosis.

All cases underwent detailed history taking, clinical examination, and investigations as per the proforma. Patients were grouped according to age, gender, residential area, severity, and level of asthma control. Children were classified as having mild asthma (intermittent and mild persistent asthma) or severe asthma (moderate persistent and severe persistent asthma). Patients already on treatment were categorized according to asthma control as controlled (well controlled) or uncontrolled (not well controlled and very poorly controlled).

All patients were initially screened using intradermal skin testing, chest radiography, absolute eosinophil count, and measurement of total serum IgE levels, along with detection of Aspergillus fumigatus-specific IgE and IgG in blood. In patients diagnosed with ABPA, cystic fibrosis was ruled out based on family history, clinical features suggestive of CF, and sweat chloride testing.

For intradermal testing, Aspergillus fumigatus antigen (1:500 weight/volume concentration) was obtained from ALCIT India Pvt. Ltd. All antihistamines and bronchodilators were discontinued 24 hours prior to testing. Buffered saline was used as the negative control, while histamine acid phosphate (1:10,000) served as the positive control. Reactions were read after 20 minutes, and intensity was assessed based on wheal size and erythema. Reactions above 1+ were considered positive, with 3+ and 4+ indicating higher degrees of sensitivity.

A chest X-ray was considered positive if it demonstrated radiological abnormalities suggestive of allergic airway disease, including transient infiltrates or fleeting opacities, gloved-finger and tramline appearances, pseudohilar lymphadenopathy, hyperinflation, lobar or segmental collapse, central bronchiectasis, and parallel-line or ring-shaped shadows.

An absolute eosinophil count greater than 300 cells/µL was considered significant. Serological assays for total serum IgE, Aspergillus fumigatus-specific IgE, and specific IgG were performed in the Department of Microbiology, Postgraduate Institute of Medical Education and Research (PGIMER), Chandigarh, using commercially available fluorescent enzyme immunoassay kits based on the Phadia principle (Thermo Fisher Scientific Inc., Uppsala, Sweden). The cutoff values were 500IU/L for total serum IgE, 0.35 kUA/L for Aspergillus-specific IgE, and 26.9 mgA/L for Aspergillus-specific IgG.

Aspergillus sensitivity (AS) was determined based on intradermal skin test results and Aspergillus-specific IgE levels.

CT scanning was performed in patients who fulfilled all other criteria for ABPA according to the Rosenberg–Patterson criteria(7), to confirm parenchymal changes detected on chest X-ray.

**Statistical analysis**: Descriptive and analytical statistical analyses were performed using SPSS software (version 17.0 for Windows; SPSS Inc., Chicago, IL, USA). Continuous variables such as eosinophil counts and IgE levels were expressed as mean ± standard deviation (SD) or median with interquartile range (IQR), depending on distribution. Comparisons between groups were performed using Student's t-test or analysis of variance (ANOVA) for normally distributed data and the Mann–Whitney U test for non-normally distributed data. Categorical variables were expressed as proportions, and differences between groups were assessed using Fisher's exact test. A p-value <0.05 was considered statistically significant.

## Results

Out of the 200 children with bronchial asthma included in the study, 36.5% were aged 1–5 years, 29.5% were 6–10 years, and 34% were 11–15 years. Females and males constituted 56.5% and 43.5% of the study population, respectively. A total of 61% of the enrolled children resided in rural areas. Mild asthma was observed in 81% of cases, while 19% had severe asthma. Asthma was controlled in 71% of patients and uncontrolled in 29% (Table 1)

**Table 1:** Demographic data of study population

Parameter (n)		Controlled Asthma (N)	Uncontrolled Asthma (N)	p-value
Age in years	0-5 (73)	55	18	>0.05
	6-10 (59)	42	17	
	11-15 (68)	45	23	
Gender	Female (113)	91	22	>0.05
	Male (87)	51	36	
Geographical area	Rural (122)	84	38	>0.05
	Urban (78)	58	20	
Severity	Mild (162)	136	26	<0.05
	Severe (38)	6	32	

In the controlled group, 37.3% had an AEC ≥300 cells/cumm, compared to 43.1% in the uncontrolled group. Mean AEC levels were 273.56 ± 139.64 cells/cumm and 301.74 ± 152.23 cells/cumm in the controlled and uncontrolled groups, respectively. This difference was not statistically significant (p > 0.05).

Among cases with total IgE >500 IU/L, 66.7% were controlled, whereas 75.8% of those with total IgE <500 IU/L were controlled. Mean total IgE was significantly higher in the uncontrolled group compared to the controlled group (p < 0.05).

Aspergillus-specific IgE >0.35 kUA/L was detected in 4.9% of controlled cases and 17.2% of uncontrolled cases. Mean levels were significantly higher in the uncontrolled group (1.79 ± 4.91 vs. 0.28 ± 1.06 kUA/L, p < 0.05).

Twelve cases were positive for Aspergillus-specific IgG, of which 42% were controlled and 58% were uncontrolled. Among the 188 IgG-negative cases, 73%) were controlled. Mean IgG levels did not differ significantly between groups (p > 0.05) (Table 2).

**Table 2:** Investigations in the study population

Investigation	Controlled Asthma Mean ± SD (Range)	Uncontrolled Asthma Mean ± SD (Range)	p-value
AEC (cells/cumm)	273.56 ± 139.64 (78-860)	301.74 ± 152.23 (109-770)	>0.05
Total IgE (IU/L)	584.29 ± 405.5 (67-2670)	788.91 ±591.90 (134-3358)	<0.05
Aspergillus specific IgE (kUA/1)	0.28 ± 1.06 (0.01-9.86)	1.79 ±4.91 (0.01-23.60)	<0.05
Aspergillus specific IgG (mgA/L)	9.82 ± 13.02 (1.23-138)	14.93 ± 22.03 (1.3-164)	>0.05

Chest X-ray opacities were observed in 5.6% (8/142) of the controlled group and 24.1% (14/58) of the uncontrolled group, with the remaining cases showing no radiographic abnormalities. This difference was statistically significant (p < 0.05).

**Figure 1:**
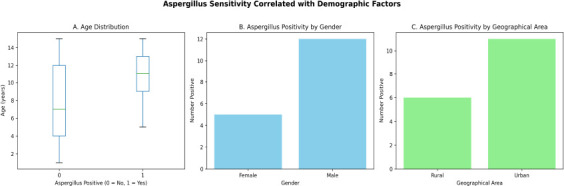
Aspergillus sensitivity correlated with demographic factors

The prevalence of Aspergillus sensitivity (AS) was 8.5%). The mean age of Aspergillus-sensitive children was significantly higher than that of Aspergillus-negative children (11.06 ± 3.13 years vs. 7.77 ± 4.27 years, p = 0.001). No significant association was observed with gender, as positivity rates were comparable between males (9.4%) and females (6.8% (p > 0.05). A significant association was found with geographical area, with urban children showing higher AS than rural children (13.9% vs. 5.0%; p < 0.05). (Figure 1)

Allergic bronchopulmonary aspergillosis (ABPA) was diagnosed in 7 children, yielding a prevalence of 3.5%). The mean age was 9.43 ±2.15 years, with most cases in the 6–10-year age group. A male predominance was observed (6 out of 7 cases). Most ABPA cases (71.4%) were from rural areas. Mild asthma was present in 5 cases, while 2 had severe asthma. Asthma control was poor in the majority, with 5 cases (71.4%) being uncontrolled.

Among ABPA-positive cases, intradermal skin test reactions were distributed as follows: 14.3% showed a 1+ reaction, 42.9% a 2+ reaction, 28.6% a 3+ reaction, and 14.3% a 4+ reaction. Differences in skin test results between ABPA groups were not statistically significant (p > 0.05).

The mean AEC was significantly higher in ABPA-positive cases (352.38 cells/cumm) compared to ABPA-negative cases (278.79 cells/cumm) (p < 0.05), indicating marked eosinophilia in ABPA (Table 3).

**Table 3:** AEC levels in ABPA positive cases

Group (N)	Mean ± SD	Median (IQR)	Range	p-value
ABPA present (7)	352.38 ±68.23	363.5 (333–395.25)	240–431	<0.05
ABPA absent (193)	278.29 ± 145.24	250 (170.5–347)	78–860	

All ABPA-positive cases had total IgE >500 IU/L, with significantly higher mean levels than ABPA-negative cases (1229.25 ± 358.12 vs. 623.12 ± 470.35 IU/L, p < 0.05). (Table 4) Among ABPA-positive cases, 85.7% had positive Aspergillus-specific IgE (>0.35 kUA/L). Mean levels were significantly higher in ABPA-positive cases (5.68 ± 5.31 kUA/L) than in negative cases (0.51 ± 2.53 kUA/L) (p < 0.05) (Table 5).

**Table 4:** Total IgE levels in ABPA positive cases

Group (N)	Mean ± SD	Median (IQR)	Range	p-value
ABPA present (7)	1229.25 ± 358.12	1181 (945.25–1450.75)	780–1924	<0.05
ABPA absent (193)	623.12 ± 470.25	524.0 (338.75–781.5)	67–3358	

**Table 5:** Specific IgE levels in ABPA positive cases

Group (N)	Mean ± SD	Median (IQR)	Range	p-value
ABPA present (7)	5.68 ±5.31	5.7 (0.96–7.09)	0.47–15.9	<0.05
ABPA absent (193)	0.51 ±2.53	0.10 (0.06–0.19)	0.01–23.6	

Forty-three percent of ABPA-positive cases had elevated Aspergillus-specific IgG, with significantly higher mean levels than ABPA-negative cases (p < 0.05) (Table 6). Among 20 probable ABPA cases, HRCT was positive in 40% (8 cases).

**Table 6:** Specific IgG levels in ABPA positive cases

Group (N)	Mean ± SD	Median (IQR)	Range	p-value
ABPA present (7)	24.55 ± 13.31	23.05 (18.77–29.35)	3.6–43.6	<0.05
ABPA absent (193)	10.75 ± 16.17	7.2 (3.9–12.45)	1.23–164	

## Discussion

In the present study, the prevalence of AS and ABPA among paediatric asthma patients was 8.5% and 3.5%, respectively. These figures lie at the lower end of the range reported in previous Indian and international studies, which have documented prevalence rates of 15–30% in children with poorly controlled asthma(8–11). This lower prevalence may be attributed to differences in study populations, as our study included all asthmatic children rather than focusing exclusively on those with poorly controlled disease. Diagnostic criteria also play a role; although the Rosenberg–Patterson criteria remain the historical standard, (7) their limitations in paediatric populations are recognized, and modified or ISHAM criteria may be more sensitive in this age group (17).

No significant association was observed between AS or ABPA prevalence and age or gender. ABPA was more common in rural children, possibly reflecting increased exposure to Aspergillus spores in agrarian regions of northern India. Most patients were residents of Punjab and surrounding areas, where environmental and climatic factors may influence sensitization rates (8).

All ABPA patients demonstrated positive skin test results. Although intradermal skin testing is not specific to ABPA, it remains a useful screening tool, especially in resource-limited settings (16).

Mean AEC and total serum IgE levels were significantly higher in ABPA-positive patients, reflecting the underlying Th2-mediated immunopathogenesis of ABPA (12). Elevated Aspergillus-specific IgE and IgG further support the diagnosis and reflect IgE-mediated hypersensitivity and immune complex-driven inflammation (14).

Chest X-ray abnormalities were present in most ABPA cases, although HRCT remains superior for detecting bronchiectasis and subtle parenchymal changes.

In summary, ABPA should be suspected in children with bronchial asthma who have persistent symptoms despite optimal therapy. Early diagnosis and appropriate corticosteroid treatment are crucial to prevent disease progression and long-term complications.

The study would have been strengthened by a larger sample size with greater representation of uncontrolled and severe asthma cases. HRCT was not performed in all patients due to concerns regarding radiation exposure and ethical considerations.

## References

[1] McIntyre A, Busse WW (2022). Asthma exacerbations: the Achilles heel of asthma care. Trends Mol Med.

[2] Global Initiative for Asthma 2024 GINA Main Report. Global strategy for asthma management and prevention.

[3] Liu AH, Covar RA, Spahn JD, Sicherer SH, Kliegman R, Staton B, St Gerne J, Schor N, Behrman R (2024). Childhood Asthma. Nelson Textbook of Pediatrics.

[4] Federico MJ, Denlinger LC, Corren J, Szefler S J, Fuhlbrigge AL (2021). Exacerbation-Prone Asthma: A Biological Phenotype or a Social Construct. J Allergy Clin Immunol Pract.

[5] Zemedkun K, Woldemichael K, Tefera G (2014). ‘Assessing control of asthma in Jush, Jimma, South West Ethiopia’. Ethiopian Journal of Health Sciences.

[6] Zhang Z, Reponen T, Hershey GK (2016). Fungal Exposure and Asthma: IgE and Non-IgE-Mediated Mechanisms. Curr Allergy Asthma Rep.

[7] Farrant J, Brice H, Fowler S, Niven R (2016). Fungal sensitisation in severe asthma is associated with the identification of Aspergillus fumigatus in sputum. J Asthma.

[8] Rosenberg M, Patterson R (1977). Clinical and immunologic criteria for the diagnosis of allergic bronchopulmonary aspergillosis. Ann Intern Med.

[9] Singh M, Das S, Chauhan A (2015). The diagnostic criteria for allergic bronchopulmonary aspergillosis in children with poorly controlled asthma need to be reevaluated. Acta Paediatrica.

[10] Agarwal R, Muthu V, Sehgal IS (2023). Aspergillus Sensitization and Allergic Bronchopulmonary Aspergillosis in Asthmatic Children: A Systematic Review and Meta-Analysis. Diagnostics (Basel).

[11] Shah A, Kunal S (2017). A review of 42 asthmatic children with allergic bronchopulmonary aspergillosis. Asia Pacific Allergy.

[12] Chetty A, Bhargava S, Jain RK (1985). Allergic bronchopulmonary aspergillosis in Indian children with bronchial asthma. Ann Allergy.

[13] Kumari J, Jat KR, Lodha R, Jana M, Xess I, Kabra SK (2020). Prevalence and Risk Factors of Allergic Bronchopulmonary Aspergillosis and Aspergillus Sensitization in Children with Poorly Controlled Asthma. J Trop Pediatr.

[14] Singh M, Chakrabarti A, Verma M, Chauhan A (2018). Clinico-Radiological Correlation in Allergic Bronchopulmonary Aspergillosis (ABPA) detected in children with poorly controlled asthma. American Journal of Respiratory and Critical Care Medicine.

[15] Agarwal Ritesh, Khan Ajmal, Aggarwal Ashutosh N (2011). Clinical relevance of peripheral blood eosinophil count in allergic bronchopulmonary aspergillosis. Journal of Infection and Public Health.

[16] Singh M, Chauhan A, Paul N, Sharma M, Verma M (2017). Allergic Bronchopulmonary Aspergillosis (ABPA) in poorly controlled children with Asthma. Am J Respir Crit Care Med.

[17] Heinzerling L, Mari A, Bergmann KC, Bresciani M, Bürbach G, Darsow U (2013). The skin prick test - European standards. Clin Transi Allergy.

[18] Agarwal R, Chakrabarti A, Shah A, Gupta D, Meis JF, Guleria R, Moss R, Denning DW, ISHAM Working Group (2024). Revised ISHAM-ABPA working group clinical practice guidelines for diagnosing, classifying and treating allergic bronchopulmonary aspergillosis/mycoses. Mycoses.

